# Remarks on Insanity

**Published:** 1818-09

**Authors:** George Parkman

**Affiliations:** Boston, N. America.


*For the London Medical and Physical Journal.*

### *Remarks on Insanity; communicated* by George Parkman, M. D. of Boston, N. America.

*(Concluded from our last.)*

TO ascertain existence of insanity or feint of it, unless the examiner's object be hid, the inquiry must be extensive.

A physician in this town asked me to see a female in his house, whose conduct was strange. He doubted whether her strangeness was involuntary, or assumed since some domestic crosses. I went to her chamber on pretence of seeing the house with a view of hiring it. Being left with her, I asked why she put her hands to her head? "She believed she had lost her head!" From such expressions, and from her general air, I concluded she was insane or knew how to feign insanity. The first was true.

*Restraints.*—When restraints are easy and exclude idea of escape; irritation, revenge, and despair, consequent to painful restraint, are prevented, the sufferer is impressed with the advisableness of submission, and with respect for directors able to accomplish judicious measures. Each patient's situation and relations in life are to be considered. Whatever peculiar measures are advisable are to be executed with a persuasively-commanding tone of conscious power, and without a chance of exciting ludicrous ideas.

Patients, who generally cause but little trouble, not unfrequently experience most severe paroxysms, almost periodically, or on unusual occurrences, or even when nothing appears to which they seem attributable. Mrs. ***** had in four months two uninterrupted raving and muttering deliriums. The first occurred on return from a walk, lasted a week, and threatened immediate fatality. She refused food and clothing. The second lasted four days. I staid much with her, keeping by me a glass of wine-whey. At moments *when her delirium was so wandering that her attention did not rest even on the pressing subject* of her delusion or disease, *inappetency of food*, I put the whey to her lips; she inadvertently swallowed: this was repeated as occasion presented, till her paroxysm subsided.*

Only a small proportion of insane persons show *permanent severe* excitement. But such cases as Mrs. *****'s, and many others, need singly, at periods, the patient and undivided attention of a person of the fullest knowledge of such cases, to qualify him for trying to alleviate such pressing evils, and for devising means of preventing immediately-threatening ones.

To such cases general directions are inadequate; com-

---

* Had she died, her friends would have received but little satisfaction from knowing that pleasant food had been put before her at regular intervals; or that her struggles against food were overcome by coercion. The violent efforts of a long paroxysm are often fatal alone, however successful single efforts may be to subdue, by coercion, disposition to fasting.

Cases like Mrs. *****'s seem exceptions from those in which, in inappetency, the system is offended by food, as by a foreign uncongenial body. Cases like her's seem analogous to the common ones of slight exhaustion after exertion; no food is craved, yet revival follows a mildly-stimulating draught.

The *general* exhaustion after even short violent exertions without recruiting food, is such, that sudden death not unfrequently occurs even at the moment of such exertion, and even in very strong persons if they are raving.

Inordinate action is generally followed by corresponding reaction. If the action be as great as the vital forces are capable of, reaction is prevented.

Furious insanity must be fatal, unless the excitement can be so modified, that it reach not its utmost limits, nor be followed by reaction beyond the sustaining powers.

bined circumstances like those in Mrs. *****'s case may not recur. The treatment is suggested, at the moment, by ever-varying circumstances, which cannot be foreseen; some of which, though unnoticed by an inexperienced observer, seem to involve the sufferer's safety, and attract the notice of the ready medical attendant. Even such a person, after many fatigues and perils, will often fail of success. But from his concentrated and unembarrassed exertions only are we to expect happy issues under such pressing and recurring difficulties.

Properly-constructed double rooms for each patient present most efficient and easy means of requisite general restraint and quiet. In Mass. Asylum the window-sashes are ash,—glass, 6 in. by 8, 24 panes,—stiles and top-rail 2 in. sq.—bottom rail 2¾ by 2,—meeting-rail 1½ by 2,—intermediate bars 2 by 1. Upper sash falls, lower sash rises 7 inches. Room-doors are fastened by a mortice or box-lock or bolt, kept in its place by a spring, and moved by an arm attached to the tumbler, which is turned by a square socket perforating it to receive the key; the box is 3 in. sq. ⅞ in. thick.

I know no sure proper means of confining the upper extremities of ingenious patients, perseveringly impatient of restraint, but a canvas straight-jacket, the lower parts of the arm-pockets sewed very strongly, the back held together by buckles and wide straps, the bottom secured round each thigh by a strap or string.

The following means may be exactly proportioned to extravagance or resistance, and are less heating and irritating. To confine the fingers, thick leather mittens without thumbs, the palms covered with sole-leather, the mittens secured round the wrist by a strong strap, with a metallic ring, through which a cord may pass, to hold the hands behind.—In sitting and lying, the hands are kept in front by a cord passing through a staple in the floor near the feet.—To confine the ankles, similar straps, the cord through the rings passing through the staple.—To confine the breast, a strap round it and the back of the chair.—To confine the thighs, a strap round each of them and one of the chair's hind legs. The chair is fixed.

*Medical Treatment*.—Of the action and state of the brain and nerves during life, as of many internal parts, we know but little. Their minute structure shows their functions but imperfectly. The time of commencement of morbid effects and peculiarities apparent on dissection cannot often be known, nor the connexion between them and insanity, nor whether many of them have not taken place since death, or are consequences of previous affections not now apparent. Insanity, like other diseases, sometimes leaves no visible traces after death.

In what part of the system disease begins, in any ailment, we seldom know. Its primary symptoms are seldom noticed. It first excites attention generally by several coexisting phenomena interfering with the individual's comfort, and apparently emanating from parts remote from each other. If intellectual "derangement" be a primary affection, but little can be known from the sufferer's account of its origin. When bodily ailment precedes insanity, we seem to have more light at first: but we cannot, from the sufferer's experience, trace the steps by which physical ailment blends itself with intellectual.

In a system intimately connected, affections are participated according to each part's susceptibility. Some parts, remote from the apparent or supposed original seat of the morbid action or affection, are sometimes more pressingly disordered, and seem first to ask aid. The healing art consists often in seizing these indications and relieving such parts: renovation is thus participated through the system, and satisfactory results follow the judicious physician's labour, characterized by energy and patience, though often by doubt. Health consists in the due action of each bodily organ; what plan is so likely to cure disease as that which tries to restore each organ to its accustomed action, in the order of apparent severity of its respective ailment? The records of five lunatic asylums give together 5351 admissions, 2792 cures. Even idiots have acquired or recovered some reason after a steady course of judicious discipline; many such cases seem attributable to baneful habits, or mismanaged insanity.

Few bodily ailments presented in insanity seem essential to it. They are as various as the symptoms of other disorders, and resemble them.

Medical applications should be made in a manner least likely to obstruct the system's tendency to spontaneous changes, generally salutary, to which physicians are often indebted for "the triumphs of art." Imitations are attempted of these changes. We know but their external marks or their effects. Our production of some appearances, like nature's, may be mistimed, injurious, and is not likely to be attended with such combination of circumstances as precede natural changes, and without which similar effects are not to be expected.

The disease may prove very long and violent; the physical powers may be exerted by it to their utmost, though they may seem equal to great exertions. I have been told by patients' relations, "we have no hope of relief for our friend, but from a process by which his maniacal vigour shall subside under general prostration."—"Diseases often seem changed, suspended, or cured, specially in their early stages, by agents producing powerful effects of any kind on the habit. Before introducing this new state of things, we should consider well whether it may not be more immediately dangerous than that to which it is opposed;—*Naturam expelles, usque non semper recurret.*" This is the province of the enlightened and experienced physician, who alone can perceive nature's feeble and almost-stifled indications; wait patiently the result of her suppressed efforts; judiciously essay artificial treatment, when she seems irrecoverably prostrated; and unbiassedly stand ready to observe the *juvantia et lædentia*, to meet his perplexities *pro re natâ*. See also "Management of Lunatics, &c." p. 22.

Daily discharge from the bowels seems indispensable to good success: for this, laxatives seem best; they leave the bowels a chance of resuming their functions. Inordinate action of the bowels induced by purges is followed by corresponding inaction; this renders necessary a repetition of the purge, &c.

If, under use of mercury as a laxative, mercurial action appears, its worth as a remedy will be known. I have seen thin persons, one of them insane, emaciate irrecoverably under unsuccessful attempts at salivation.

The most useful consequences of mercury have appeared, when the patient has been *unexpectedly* salivated; i. e. when he was not so diseased as to have lost *quick* susceptibility of salivation. Sometimes in severe disease, mercury, emetics, cathartics, &c. apparently indicated, and assiduously administered, fail of their usual sensible effects. The patient's safety seems to depend on restoration of this susceptibility, by art or nature's efforts.

A labourer, under erysipelatous inflammation of his face, his eyes and ears closed, was uninterruptedly delirious, fancying himself lifting heavy bales, made corresponding exertions, and sweated profusely. Thinking this would soon exhaust him, I thought it necessary to change the train of his ideas. Thinking nothing likely to do this but a most vivid physical impression, I produced a great irritation on his crown; he did not recur to his delusion, and became in a situation to receive remedies to which his restoration seemed owing.

A slender female, uninterruptedly delirious, constantly stamped with her feet. To interrupt this exertion, threatening to enfeeble her irrecoverably, I advised blisters to her soles. I have given similar advice, when this seemed preferable to other restriction, for patients disposed to run away, kick, or strike.

Much advantage has been attributed to the warm-bath, at 82° to 88°, combined with the *douche;* i. e. the patient, under excitement, being confined in the warm-bath, by a wooden cover to the tub, with a notch for his neck; a stopcock from a reservoir above is turned; so cold water falls on his head by drops, or in a small stream. The falling water is generally an object of terror. Perhaps a vapour-bath, described in the London Med. and Phys. Journal, No. 25, may serve for the warm-bath.

Some foreign medicines used in insanity have been shamefully adulterated; others, even of domestic origin, mistaken. Hence, and from the diversity of disease, and of the physical and moral management, arise some discordant accounts of medicines.

---

*For the London Medical and Physical Journal.*

